# Effect of Three Semen Extenders on Sperm Quality and In Vitro Fertilization Rates of Fresh and Cryopreserved Sperm Collected from Llama (*Lama glama*) Vas Deferens

**DOI:** 10.3390/ani14111573

**Published:** 2024-05-25

**Authors:** Manuel G. Pérez-Durand, Carlos W. Bustamante, Pedro P. Machaca, Wilber García, Eloy A. Condori, Rassiel Macedo, Eliseo Fernández, Yan P. Manrique, Miguel A. Gutiérrez-Reinoso, Uri H. Perez-Guerra, Manuel García-Herreros

**Affiliations:** 1 Facultad de Medicina Veterinária y Zootecnia, Universidad Nacional del Altiplano, Puno 21001, Peru; mgperez@unap.edu.pe (M.G.P.-D.); epfernandez@unap.edu.pe (E.F.); yan.manrique@unap.edu.pe (Y.P.M.); 2 Facultad de Ciencias Agrárias, Universidad Nacional San Antonio Abad del Cusco, Cusco 08000, Peru; carlos.bustamante@unsaac.edu.pe (C.W.B.); eloy.condorich@unsaac.edu.pe (E.A.C.); rassiel.macedo@unsaac.edu.pe (R.M.); 3 Facultad de Ciencias Agropecuárias, Escuela Profesional de Medicina Veterinária y Zootécnia, Universidad Jorge Basadre Grohmann, Tacna 23000, Peru; pedro.mac20@gmail.com; 4 Facultad de Medicina Veterinária, Instituto Veterinário de Investigaciones Tropicales y de Altura, Universidad Nacional Mayor de San Marcos, Maranganí 08258, Peru; wgarciav20@unmsm.edu.pe; 5 Facultad de Ciencias Agropecuarias y Recursos Naturales, Carrera de Medicina Veterinaria, Universidad Técnica de Cotopaxi (UTC), Latacunga 050150, Ecuador; miguel.gutierrez@utc.edu.ec; 6 National Institute for Agricultural and Veterinary Research (INIAV), 2005-424 Santarém, Portugal

**Keywords:** llamas, sperm quality, cryopreservation, vas deferens, in vitro fertilization

## Abstract

**Simple Summary:**

Simple Summary: There are very few reports regarding Assisted Reproductive Technologies in South American camelids, including llamas (*Lama glama*). The sperm quality and in vitro fertilization rates from fresh and cryopreserved sperm collected from vas deferens using different preservation extenders were investigated in llama males as a model for its application in other South American camelids. The obtained results from our research showed that the preservation extender was a determining factor in significantly improving the in vitro fertilization rates when using fresh and cryopreserved sperm samples obtained from the vas deferens in llamas (*Lama glama*).

**Abstract:**

The advances in Assisted Reproductive Technologies (ARTs) applied in South American camelid species are still scarce. The aim of this study was to compare the effects of three semen extenders, before and after the cryopreservation of spermatozoa obtained from the vas deferens, on sperm quality parameters and in vitro fertilization rates of llama (*Lama glama*) oocytes. Mature fertile llama males (*Lama glama*; *n* = 6; age: 48–60 mo.; BCS: ~2.7) were included in the study. Sperm samples were collected from each male using the surgical technique of the vas deferens deviation. Then, the sperm samples were pooled and diluted with the Tris-EY, Andromed^®^, or BioxCell^®^ extender in order to subsequently carry out the sperm cryopreservation process. The sperm quality assessment related to each extender was performed before and after cryopreservation with regard to sperm morphological abnormalities, acrosome integrity, sperm viability, membrane permeability, and sperm motility traits. Moreover, in vitro fertilization (IVF) procedures were carried out to evaluate the in vitro fertility of the cryopreserved sperm samples using each extender. Overall, significant differences were observed before and after cryopreservation regarding acrosome integrity, sperm viability, membrane permeability, and sperm motility traits among the extenders used, where Tris-EY and Andromed^®^ were better than BioxCell^®^ (*p* < 0.05); however, no differences were observed regarding the sperm morphological abnormalities among extenders (*p* > 0.05). Moreover, multiple differences were observed with regard to the velocity and linearity kinematic parameters obtained by computerized analysis before and after the cryopreservation process, irrespective of the extender used (*p* < 0.05). Finally, differences were observed regarding the in vitro fertilization rates among the different extender-derived samples (*p* < 0.05). In conclusion, the sperm quality using Tris-EY and Andromed^®^ was better before and after cryopreservation compared to that using BioxCell^®^. Although the number of fertilized oocytes obtained after the IVF process between Tris-EY and Andromed^®^ was similar, Andromed^®^-derived samples showed the best sperm quality results before and after cryopreservation. This indicates that the cryopreservation extender is a determining factor in significantly improving in vitro fertilization rates when using sperm samples obtained from vas deferens in llama (*Lama glama*) males.

## 1. Introduction

Semen collection and sperm processing in South American camelids have important limitations due to the particular species-specific semen characteristics, which are frequently viscous, stringy, and sometimes foamy, making it difficult for in vitro processing and its application in different sperm quality determination procedures [1,2,3,4,5]. For these reasons, the application of artificial insemination (AI) in South American camelids is not as widely used as in other domestic species [6,7]. Sperm preservation in South American camelids has several advantages related to the transference of genetic material at low costs, and this fact could improve the development of artificial insemination in different camelid species [8]. However, the development of this biotechnology in camelids includes the study of various procedures, such as semen collection, handling, and cryopreservation [9,10,11,12].

Sperm extenders are usually based on biological compounds whose function has been, among others, to avoid sudden pH changes, to improve the energy source, to increase the tolerance to chilling and cryopreservation, and to avoid damage or oxidative stress during the cryopreservation process [13]. The use of commercial extenders has been characterized by ease of preparation (avoiding the addition of egg yolk) and avoiding additional specific equipment for sperm sample processing [14]. Another advantage of commercial extenders is that they allow repeatable results [15]. On the other hand, the most commonly used non-commercial extender in South American camelids has been the Tris (hydroxymethyl aminomethane) based extender supplemented with egg yolk as a sperm membrane protector and glycerol as a cryoprotectant during the cryopreservation process [16,17]. The routine assessment of sperm viability has been characterized by determining motility, membrane integrity, and acrosome integrity, among others, including several DNA evaluation techniques [18,19,20]. In camelids, several extenders containing animal- or plant-origin proteins have been investigated previously in fresh, refrigerated, or cryopreserved sperm [1,3,6,11,14,15,18,19,20]. Tris-EY is an egg yolk-based extender that has been commonly used in camelids [1,14,15,20], whereas BioxCell^®^ is a soybean lecithin-based extender that has only been used in alpacas [1]. On the other hand, Andromed^®^ is another soy lecithin-based extender that has only been used in fresh and refrigerated sperm samples from alpacas and llamas [3,6,11,15]. However, no studies have been carried out on vas deferens-derived sperm samples and the effects of these extenders on in vitro fertility based on IVF assays. Therefore, it would be necessary to carry out more in-depth studies on fresh and cryopreserved sperm samples recovered from the vas deferens in llamas and the impact of the Tris-EY, Andromed^®^, and BioxCell^®^ extender on sperm quality and IVF results.

Recently, the evaluation of sperm motility using computerized-aided sperm analysis systems has been applied in South American camelid species, increasing the objectivity of sperm kinematic parameters [21]. The use of new methodologies for the analysis of sperm quality of little-known species has become necessary, as is the case in South American camelids, always with the aim of using the new information obtained for application in different reproductive biotechnologies, such as in vitro fertilization (IVF) [22]. The use of new Assisted Reproductive Technologies (ARTs) in South American camelids, such as IVF, allows new information to be obtained regarding the gamete and embryo physiology for the subsequent application of developing ARTs in different South American camelid species [22,23,24]. However, these types of technologies require a series of processes, such as oocyte retrieval, oocyte maturation, sperm capacitation, in vitro fertilization, and in vitro embryo culture, among others [25]. Currently, these types of technologies are being used by different researchers in order to develop more successful protocols, although most of the trials carried out have been considered experimental in order to generate a suitable protocol for application in different South American camelid species [26]. Recently, our research team has obtained morula and blastocyst percentages that vary between 17.4% and 16.5%, respectively, at high altitude environmental conditions, using oocytes obtained from ovaries collected in slaughterhouses, which have been recovered using follicular aspiration techniques similar to those used in cattle [27,28].

Thus, the main objective of the present study is to compare the effect of different extenders on sperm quality traits during the cryopreservation process in order to determine the potential use of cryopreserved sperm samples recovered from vas deferens in in vitro fertilization procedures in llamas (*Lama glama*) as a model to study other South American camelid species.

## 2. Materials and Methods

### 2.1. Ethical Statement

The present research was conducted according to the guidelines of the Declaration of Helsinki and following the Code of Ethics for animal experiments, as reflected in the ARRIVE guidelines available at http://www.nc3rs.org.uk/ARRIVEchecklist (accessed on 1 December 2023). This study was approved by the Bioethics Committee for the use of experimental animals at the Universidad Nacional del Altiplano (Puno, Perú; Approval Date: 17 January 2020, Code Number: DE-003866-2019).

### 2.2. Experiment Location, Reagents, and Media

This study was conducted in the Laboratory of Animal Reproduction of the Faculty of Veterinary Medicine and Animal Husbandry at the Universidad Nacional del Altiplano Puno (Perú) (latitude: −15.82435° W 15°49′28″ S; longitude: −70.01573°/70°0′57″ at 3827 m.a.s.l.). All reagents and media used in the experiments, unless otherwise stated, were of analytical grade and purchased from Sigma-Aldrich Chemical Company (St. Louis, MO, USA).

### 2.3. Animals and Procedure of Sperm Sample Collection from Vas Deferens

Six healthy reproductively mature fertile and genetically heterogeneous llama (*Lama glama*) males (*n* = 6; Age: 48–60 mo.; B.W.: 120–140 kg.; B.C.S.: ~2.7) were used [29]. All animals were bred in the Faculty of Veterinary Medicine and Animal Husbandry and kept in isolated enclosures to avoid herd conditioning effects on semen quality. The males were maintained under the same nutritional (standard diet, natural pastures, and water ad libitum) and environmental conditions (outdoor access) during all the experiments. Two sperm samples per male (a total of twelve samples) were collected and pooled per day to obtain homogeneous samples. These sperm samples were collected from each male on consecutive weeks (twice a week) over three months (January, February, and March) using the surgical technique of the deviation of the vas deferens described by Perez et al. [30]. The first collection of sperm samples was performed two months after the surgical procedure. The surgical procedure of the vas deferens consisted of several steps. First of all, the males were fasted for 24 h, and then they were administered 0.1 mg/kg B.W. of acepromazine maleate. All males were placed in a dorsal recumbency position, and the surgical field (inguinal region) was prepared using local anesthesia. Then, a small cut (4 cm) was made above the penis, and the vas deferens (right and left side) were located and dissected (length: 7 cm). The dissected vas deferens were redirected underneath the subcutaneous tissue and fixed to the skin of the femoral region, protected with a temporary patch. The llama males were previously immobilized, knocked down, and placed in lateral recumbency. Then, prior to each sperm sample collection, the fistulas (externalized vas deferens) were cleaned using distilled water. In order to obtain the sperm samples, it was necessary to rub (linear friction) the vas deferens with the fingertips towards the exit of the fistula (located on the inner thighs). The spermatozoa (spz) were obtained as the sample drops appeared at the edge of the fistula (~10 µL per vas deferens) using a micropipette (Micropipette, Boeco^®^, Hamburg, Germany) and diluted with 1 mL of the extender (Tris-EY, Andromed^®^ or BioxCell^®^) according to the case. The sperm samples were collected from all males and pooled to avoid any potential male effect. The sperm samples were kept at ~37 °C until transfer to the laboratory.

### 2.4. Sperm Quality Assessment

#### 2.4.1. Sperm Concentration Determination

Raw sperm samples were collected from llama (*Lama glama*) vas deferens. After sperm collection, the pooled samples were kept at 37 °C in a water bath. The sperm concentration in each pooled sample was performed using the Neubauer hemacytometer (Marienfeld, Lauda-Königshofen, Germany). First, the sperm sample was aspirated and suspended in bi-distilled water (1: 200 *v*/*v*). Subsequently, a sample was placed in the Neubauer hemacytometer and evaluated under the inverted microscope (Leica, Leica Microsystems CMS GmbH, Mannheim, Germany) using the 200× magnification objective. The sperm concentration was determined by counting 5 squares randomly.

#### 2.4.2. Sperm Morphology Assessment

Sperm morphological abnormalities were determined using an aliquot of the pooled sperm sample using phase-contrast inverted microscopy (Leica Microsystems CMS GmbH, Mannheim, Germany) under a 400× magnification objective. Briefly, 10 µL of sperm samples and 20 µL of the solution (1% formaldehyde solution in phosphate-buffered saline (PBS)) were suspended until a sperm suspension of 10 × 10^6^ spz/mL was obtained. Then, 10 µL of the sperm suspension was placed on a tempered slide. Duplicate smears were performed, and at least 200 spermatozoa per smear were analyzed.

#### 2.4.3. Acrosome Integrity Analysis

Similarly to sperm morphology evaluation, the sperm acrosome integrity was determined using an aliquot of the pooled sperm sample using phase-contrast inverted microscopy (Leica Microsystems CMS GmbH, Mannheim, Germany) under a 1000× magnification objective. Briefly, 10 µL of the sperm sample and 20 µL of the solution (3% glutaraldehyde solution in phosphate-buffered saline (PBS)) were suspended until a sperm suspension of 10 × 10^6^ spz/mL was obtained. Then, 10 µL of the sperm suspension was placed on a tempered slide. Duplicate smears were performed, and at least 200 spermatozoa per smear were analyzed.

#### 2.4.4. Sperm Membrane Integrity Evaluation

Sperm vitality was evaluated using phase-contrast optic microscopy by assessing the sperm membrane integrity using eosin and nigrosin staining [31]. Briefly, 10 µL of the sample and 20 µL of dye (sperm suspensions of 10 × 10^6^ spz/mL) were placed on a tempered slide. Then, duplicate smears were performed. Spermatozoa were then examined under a Leica phase-contrast microscope (Leica Microsystems CMS GmbH, Mannheim, Germany) under a 40× magnification objective. Red-stained (eosin-positive) spermatozoa were considered viable cells, whereas dark-stained (nigrosin-positive) spermatozoa were considered non-viable cells. At least 200 spermatozoa per slide were analyzed, as described by Gomez-Quispe et al. [32].

#### 2.4.5. Sperm Membrane Permeability Analysis

The Hypo-Osmotic Swelling test (HOST) was used for sperm membrane permeability assessment. A hypo-osmolar solution composed of citrate dihydrate (0.735 g) and fructose (1.351 g) diluted in 100 mL of bi-distilled water was used following the recommendations by Flores Huarco et al. [33]. Sperm evaluation was carried out considering those spermatozoa that showed swelling at the tail level (functional membrane permeability) as positive. At least 200 spermatozoa per sample were analyzed, as recommended by Gomez-Quispe et al. [32].

#### 2.4.6. Sperm Motility Assessment

Sperm motility evaluation was performed before and after the cryopreservation process. Progressive individual motility scores were assessed under a coverslip (18 × 18 mm) on a warm stage (37 °C) by phase-contrast-inverted microscopy (10× objective) examined under a Leica microscope (Leica Microsystems CMS GmbH, Mannheim, Germany) after a dilution (1:100) in each extender (Tris-EY, Andromed^®^ or BioxCell^®^, pH = 7.4, maintained at 37 °C). Motility was evaluated by measuring the following parameters: progressive motility (PM), oscillatory motility (OM), circular motility (CM), and total motility (TM), which is the sum of the previous parameters [6,15,21].

The objective motility assessment was performed using a commercially available computer-assisted semen analysis system (CASA; AndroVision^®^, Minitube, Tiefenbach, Germany) equipped with a phase-contrast microscope using a 10× negative contrast. The video signal was acquired using a digital camera attached to the microscope and its respective software (AndroVision^®^ software, Minitube, Tiefenbach, Germany) for sperm motility analysis. In addition, digitized images were made up of 1,920,000 pixels (picture elements) and 256 gray levels for sperm functionality analysis, where the recorded videos were analyzed at 25 frames per second. At least 125 spermatozoa per field were randomly captured in duplicate in the acquisition mode of the software. Each sperm kinetic was measured for twelve primary kinematic parameters as follows: curvilinear velocity (VCL; µm/s), as the average path velocity of the sperm head along its true trajectory per unit time; straight-line velocity (VSL; µm/s), as the average path velocity of the sperm head along a straight line from its first to its last position per unit time; average path velocity (VAP; µm/s), as the average velocity of the sperm head along its average trajectory per unit of time; distance curved line (DCL; µm), as the distance that the sperm head moves per time unit on the curve path; distance straight line (DSL; µm), as the distance that the sperm head moves per time unit on the straight path; distance on average path (DAP; µm), as the distance that the sperm head moves per time unit on the average path; amplitude of lateral head displacement (ALH; µm), as the average value of the extreme side-to-side movement of the sperm head in each beat cycle; head cross frequency (BCF; Hz), as the frequency at which the actual sperm trajectory crosses the average path trajectory; head activity (HAC; rad), as the average of all calculated differences of the mean axis angle of two consecutive frames; wobble coefficient index (WOB; %), as the ratio between VAP and VCL (×100); linearity index (LIN; %), as the ratio between VSL and VCL (×100); and the straightness index (STR; %), as the ratio between VSL and VAP (×100). The measurements of individual sperm kinetics from each sample were saved in an Excel v. 2019 (Microsoft Corporation, Redmond, WA, USA) compatible database by the software for further analysis.

### 2.5. Extender Preparation: Tris-EY, Andromed^®^ and BioxCell^®^

Tris-EY extender was prepared every week using Tris (tris-hydroxymethyl-aminomethane; 2.42 g), citric acid (1.34 g), fructose (1.25 g), egg yolk (20%), and glycerol (5%) in 100 mL of bi-distilled water. After the addition of egg yolk, the solution was centrifuged at 4000 r.p.m. for 30 min and filtered with a paper filter of 30 µm (Grade 113, Whatman^®^, Dassel, Germany). The first sperm dilution at 37 °C (collection phase) was performed with fraction A (Tris, citric acid, fructose, and egg yolk), and the second dilution was performed at 5 °C (equilibration phase) with fraction B (Tris, citric acid, fructose, egg yolk, and glycerol). The Andromed^®^ (Minitube GmbH, Tiefenbach, Germany) extender [composition: Tris, citric acid, phospholipids, fructose, glucose, antioxidants, pH buffers, glycine, soy lecithin: 1%—1 g/L; glycerol: 6.7%—38.47 g/L; antibiotics (Tylosin: 0.57 g/L, gentamicin: 0.286 g/L, Spectinomycin: 0.343 g/L, Lincomycin: 0.172 g/L); bi-distilled ultrapure water] was prepared on the same day of sperm collection using a 1:4 ratio (extender: bi-distilled water). Briefly, 4 mL of bi-distilled water was placed in a graduated tube, and 1 mL of Andromed^®^ was added. Then, the dilution was kept at 37 °C until use during the sperm collection phase. The second dilution at 5 °C was carried out using the Andromed^®^ extender at the same temperature (equilibration phase). The BioxCell^®^ (IMV Technologies, L’Aigle, France) extender (composition; Tris: 2.3 g/L; Sodium citrate: 6.2 g/L; Potassium chloride: 0.8 g/L; fructose: 1.2 g/L; Monohydrate lactose: 0.8 g/L; glycine: 0.2 g/L; anhydrous glucose: 0.5 g/L; Taurine: 0.005 g/L; gentamicin sulfate: 0.24 g/L; Tylosin tartrate: 0.33 g/L; Linco-Spectin 100: 0.383 g/L; glycerol: 7%—40.2 g/L; Hydrate of calcium lactate: 0.7 g/L; soy lecithin: 1.5%—1.5 g/L; Monohydrate citric acid: 2.5 g/L; bi-distilled ultrapure water) was prepared on the same day of the sperm collection in a similar to the Andromed^®^ extender using a 1: 4 ratio (extender: bi-distilled water). Then, the dilution was mixed and kept at 37 °C until use during the sperm collection phase. The second dilution at 5 °C was carried out using the BioxCell^®^ extender at the same temperature (equilibration phase).

### 2.6. Sperm Cryopreservation Process

For the sperm cryopreservation process, the sperm sample pools were diluted in each extender to ~120 × 10^6^ spz/mL and subjected to the cooling phase (5 °C) for 150 min. In the case of the Tris-EY extender, fraction B was added at 5 °C. Regarding Andromed^®^ and BioxCell^®^, the extender volume was adjusted and added until a proper concentration at 5 °C was reached. All sperm samples were kept at 5 °C for 30 min. (equilibration phase) irrespective of the extender used. The total duration of the cryopreservation process (cooling phase + equilibration phase) was 180 min. Subsequently, the sperm samples were stored in 0.25 mL straw (IMV Technologies^®^, L’Aigle, France) and cryopreserved in a semen bio-freezer device (TK3000 CSE, TK Technologies^®^, São Bernardo Do Campo, Brazil) at a freezing rate of −20 °C per min. (total 6 min) until the temperature of −120 °C was reached, as recommended by Perez et al. [20]. Then, the sperm samples were stored in liquid nitrogen at −196 °C. Finally, for the evaluation of the cryopreserved sperm samples, the straws were thawed at 37 °C for 45 s in a water bath before the quality and the fertility potential evaluation.

### 2.7. In Vitro Fertilization Process

Llama ovaries were collected from a slaughterhouse and transported to the laboratory in a thermos within 4 h after collection. The ovaries were kept in a physiological solution (NaCl 0.9%; Medifarma^®^, Lima, Peru) and 50 mg/mL of gentamicin (Gentamicina, Genfar^®^, Ate, Peru) at 32 to 35 °C. The oocyte collection and the washing process were performed, as recommended by [27], as well as the oocyte maturation process, which was performed in an incubator at 38.5 °C with 5% CO_2_ and >90% humidity for 36 h. The oocyte maturation was assessed by the presence of the first polar body. Then, groups of 10 oocytes were washed twice in FERT TALP medium [CaCl_2_·2H_2_O (29.4 mg); KCl (23.9 mg); MgCl_2_·6H_2_O (10.1 mg); NaH_2_PO_4_·H_2_O (5.5 mg); Lactic Acid (Sodium salt; 60% *w*/*w* syrup; 186 µL); NaCl (666 mg); NaHCO_3_ (210 mg); Na Pyruvate (1.0 mL); Penicillamine; Hypotaurine; Epinephrine; BSA (Fraction V; 600 mg); Gentamycin] and transferred to a drop of ~50 µL of clean FERT TALP medium [26,27,34]. Then, the in vitro fertilization process was performed using 2 µL of the sperm sample (final concentration: 1 × 10^6^ spz/mL). Gamete interaction was performed under incubation conditions for 24 h at 38.5 °C in a humid atmosphere (>90%) and 5% CO_2_. Fertilization was assessed by the identification of the second polar corpuscle at the level of the perivitelline space.

### 2.8. Statistical Analysis

All statistical analyses were performed using the R 4.3.0 software (R Core Team, 2020). All data derived from sperm concentration, morphological abnormalities, acrosome integrity, sperm viability, membrane permeability, and sperm motility traits were subjected to descriptive statistics (mean, standard error, homoscedasticity, and normality tests). The evaluated variables that fulfilled the assumptions were subjected to a completely randomized design (one-way ANOVA) and Tukey’s test, and in the case of not fulfilling the assumptions, the variables were subjected to a Kruskal–Wallis test and the subsequent post hoc test. In addition, all data from all the sperm analyzed by the computer-assisted analysis were imported into a single data set or data matrix that represented more than 5000 observations, each one defined by the kinetic descriptors specified before. The objective motility analyses were evaluated by means of a multi-analysis of variance (MANOVA) to evaluate the influence of two independent variables on the mean kinetic parameters. The level of significance was set at *p* < 0.05.

## 3. Results

### 3.1. Sperm Quality Traits in Samples Obtained from Llama (Lama glama) Vas Deferens

#### 3.1.1. Sperm Concentration

The sperm concentration in samples obtained in llama males (*n* = 30) was 41.1 ± 5.3 × 10^6^ spz/mL. No differences were observed regarding sperm concentration values after dilution using different cryopreservation extenders (*p* > 0.05).

#### 3.1.2. Sperm Morphological Defects in Cryopreserved Sperm Samples Obtained from Llama (*Lama glama*) Vas Deferens Using Different Extenders

No significant differences were observed among cryopreservation extenders regarding the total sperm defects (*p* < 0.05). Moreover, no significant differences were observed among cryopreservation extenders with regard to cytoplasmic droplets, bent/coiled tails, midpiece defects, and microcephalia percentage (*p* > 0.05); however, statistical differences were observed between Andromed^®^ and BioxCell^®^ compared to Tris-EY regarding double-tailed sperm cells with the latter showing the highest percentage (*p* < 0.05). The detached head and detached tail percentage were significantly higher in Tris-EY compared to Andromed^®^ extender samples; however, no differences were observed regarding the same parameters between Tris-EY and BioxCell^®^ sperm samples (*p* > 0.05; Table 1).

#### 3.1.3. Acrosome Membrane Integrity during the Cryopreservation Process Using Different Extenders in Spermatozoa Obtained from Llama (*Lama glama*) Vas Deferens

Significant differences were observed among cryopreservation extenders regarding acrosome membrane integrity at 37 °C and at 5 °C (*p* < 0.05). No significant differences were observed between Andromed^®^ and BioxCell^®^ at 5 °C (*p* > 0.05); however, statistical differences were observed between both and Tris-EY, with the latter being the highest at 5 °C (*p* < 0.05). Moreover, significant differences were observed regarding acrosome membrane integrity among cryopreservation steps irrespective of the cryopreservation extender evaluated (*p* < 0.05; Table 2).

#### 3.1.4. Plasma Membrane Integrity and Permeability during the Cryopreservation Process Using Different Extenders in Spermatozoa Obtained from Llama (*Lama glama*) Vas Deferens

Table 3 shows the plasma membrane integrity and plasma membrane permeability using different extenders during the cryopreservation process in sperm samples obtained from llama (*Lama glama*) vas deferens. Significant differences were observed among cryopreservation extenders regarding sperm plasma membrane integrity at 37 °C (*p* < 0.05). No significant differences were observed between BioxCell^®^ and the other extenders, (*p* > 0.05); however, statistical differences were observed between Tris-EY and Andromed^®^ with the first having the lowest and the second the highest percentage obtained when both extenders were compared (*p* < 0.05). Moreover, significant differences were observed regarding membrane permeability among cryopreservation extenders, with the Tris-EY extender being the highest and significantly different from the other extenders (*p* < 0.05). No differences were observed between the Andromed^®^ and BioxCell^®^ extenders regarding membrane permeability at 37 °C (*p* > 0.05) (Table 3).

#### 3.1.5. Sperm Motility during the Cryopreservation Process Using Different Extenders in Spermatozoa Obtained from Llama (*Lama glama*) Vas Deferens

Table 4 shows the sperm motility parameters using different extenders during the cryopreservation process in sperm samples obtained from llama (*Lama glama*) vas deferens. Significant differences were observed among sperm cryopreservation extenders regarding total motility, progressive motility, and oscillatory motility post-thawing (*p* < 0.05). No significant differences were observed among the different sperm cryopreservation extenders with regard to circular motility post-thawing (*p* > 0.05). Moreover, statistical differences were observed in Tris-EY and Andromed^®^ compared to BioxCell^®^ in all motility parameters evaluated at 37 °C and 5 °C except for progressive motility (*p* < 0.05). In general, Tris-EY and Andromed^®^ extenders showed better results in all motility parameters compared to BioxCell^®^, with the latter showing the lowest percentage in all motility parameters irrespective of the cryopreservation step evaluated. Overall, significant differences were observed between the pre-freezing (37 °C and 5 °C) steps and the post-thawing results in all motility parameters irrespective of the sperm cryopreservation extender studied (*p* > 0.05; Table 4).

Table 5 shows the sperm kinematic parameters using different extenders before and after cryopreservation in sperm samples obtained from llama (*Lama glama*) vas deferens. Significant differences were observed among sperm cryopreservation extenders regarding sperm kinematic parameters before and after cryopreservation (*p* < 0.05). No significant differences were observed among the extenders regarding VCL, DSL, ALH, HAC, WOB, and LIN parameters before cryopreservation (*p* > 0.05). Moreover, no differences were observed between Tris-EY and Andromed^®^ with regard to VSL, VAP, DCL, DAP, BCF, and STR parameters before cryopreservation (*p* > 0.05). On the contrary, significant differences were observed between Tris-EY and Andromed^®^ compared to BioxCell^®^ regarding the same parameters, with the latter presenting as the lowest for all the kinetic variables shown before (*p* < 0.05).

The Andromed^®^ extender showed the highest VCL, VSL, VAP, DCL, DSL, DAP, and BCF values compared to Tris-EY and BioxCell^®^ extenders after the cryopreservation process (*p* < 0.05); however, no differences were observed between Tris-EY and BioxCell^®^ regarding the same kinetic parameters (*p* > 0.05). No significant differences were observed between Tris-EY and Andromed^®^ with regard to ALH and HAC after cryopreservation (*p* > 0.05); however, statistical differences in the same parameters were observed when Tris-EY and Andromed^®^ were compared to BioxCell^®^, with the latter presenting as the lowest compared to the others after cryopreservation (*p* < 0.05).

Overall, significant differences were observed when the sperm samples were compared before and after cryopreservation within the same extender and within the same kinetic parameter, obtaining lower kinetic values after cryopreservation irrespective of the parameter and extender analyzed (*p* < 0.05). The exception was observed in BioxCell^®^-derived samples where WOB, LIN, and STR were significantly higher after the cryopreservation process (*p* < 0.05; Table 5).

### 3.2. In Vitro Fertilization Rates before and after Sperm Cryopreservation Process Using Different Extenders in Samples Obtained from Llama (Lama glama) Vas Deferens

**Figure 1:**
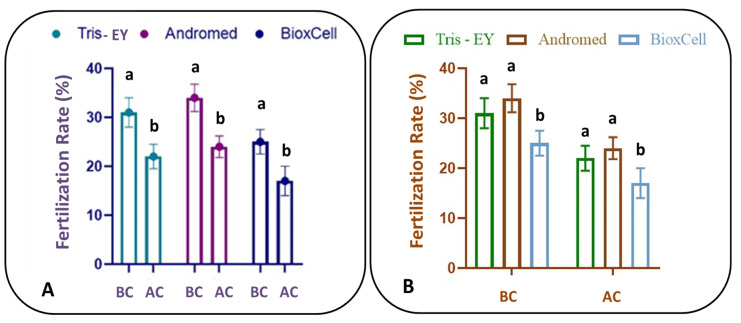
In vitro fertilization rates (%) using different cryopreservation extenders in sperm samples obtained from llama (*Lama glama*) vas deferens. (**A**) In vitro fertilization rates (%) before (BC) and after (AC) cryopreservation within each extender. Different letters (a,b) represent significant differences within each extender (*p* < 0.05). (**B**) In vitro fertilization rates (%) before (BC) and after (AC) cryopreservation among extenders. Different letters (a,b) represent significant differences among extenders before and after the cryopreservation process (*p* < 0.05).

Figure 1 and Table 6 show the in vitro fertilization rates of fresh and cryopreserved sperm samples obtained from llama (*Lama glama*) vas deferens using different extenders. In all cases, within extenders, significant differences were observed with regard to fertilization rates before and after the cryopreservation process (*p* < 0.05; Figure 1A).

Moreover, significant differences were observed among the different sperm extenders before and after cryopreservation regarding fertilization rates (*p* < 0.05; Figure 1B). No significant differences were observed between Tris-EY and Andromed^®^ regarding fertilization rates (*p* = 0.987; Figure 1B); however, there were slightly better results when using the Andromed^®^ compared with the Tris-EY extender. The lowest in vitro fertilization rate was obtained when the BioxCell^®^ extender was used before and after the cryopreservation process (Figure 1B).

## 4. Discussion

The present study investigates potential differences in sperm quality in samples obtained from the vas deferens on llamas (*Lama glama*) during the cryopreservation process and the impact of using different extenders on fertility results tested by in vitro fertilization techniques. In general, better sperm quality values were observed during the pre-freezing phase in spermatozoa, cryopreserved using Tris-EY and Andromed^®^ extenders compared to BioxCell^®^ in most of the parameters evaluated, except for plasma membrane integrity and permeability where similar results were observed between Andromed^®^ and BioxCell^®^. In general, it can be concluded that the Andromed^®^ extender could be the extender of choice to obtain the best results when applied to spermatozoa samples collected from the vas deferens in llamas. The present results in terms of sperm quality were slightly better than those observed by other authors who reported motility percentages between 56.8% and 56.2% using the commercial extender Triladyl^®^, probably due to composition differences between the different extenders [13,30]. However, all percentages observed in terms of sperm quality could be considered similar and acceptable, probably due to the fact that the buffer composition was similar, avoiding sudden changes in pH and also providing different energy sources, such as glucose or fructose [17].

Regarding sperm morpho-abnormalities, there is no information in the literature on the influence of the extenders used in the present study and the impact of cryopreservation on samples obtained from the vas deferens in llamas. Compared to other camelid species, the percentage of cytoplasmic droplets observed in alpaca studies was significantly higher compared to the present study in llamas using the same collection technique [30]. In addition, lower percentages of sperm head morpho-abnormalities were also observed in llamas compared to alpacas [30].

The sperm quality obtained in the present study was better than that reported by other authors using epididymal sample collection for sperm cryopreservation in alpacas [35,36]. The sperm motility of 14.0, 8.6 and 17.0% was observed in samples obtained from alpaca epididymis, which were maintained in a physiological buffer solution (Phosphate-Buffered Saline, PBS) and diluted using Tris, Tes, and skim milk, respectively. On the other hand, mean viability was 32.6%, and plasma membrane integrity was 34.5%. Moreover, using the artificial vagina collection method and evaluating three different extenders, Raymundo et al. [37] achieved a motility of 54.0 ± 8.0%. On the other hand, Giuliano et al. [38] reported sperm motility of 32.3 ± 20.4%, plasma membrane permeability (HOST) of 36.1 ± 13.1%, and viability of 54.1 ± 17.0% using electroejaculation in llamas, while the comparisons of total motility and oscillatory motility parameters were better in the present study except when BioxCell^®^ was used. Progressive motility was higher (26.9%) in the study by Giuliano et al. [38] compared to the results of the present study. Furthermore, considering that, in general, most of the results in the study by Giuliano et al. [38] were lower than those of the present study, it could be due to the type of semen sample collection method that can directly or indirectly influence the sperm characteristics as the ejaculates present secretions from the adnexal glands increasing the semen viscosity, frequently seen in camelids [39]. A study with the same collection method using similar extenders and under similar environmental conditions reported slightly lower percentages of total motility, oscillatory motility, and circular motility and higher percentages of progressive motility in relation to the results of the present study considering that the use of extenders, such as Andromed^®^ and Tris-EY, allowed acceptable sperm parameters to be obtained after the sperm collection from the vas deferens and the cryopreservation process [15].

In the present study, significant differences were observed when comparing the three cryopreservation extenders regarding total motility, with Andromed^®^ being the best, followed by Tris-EY and finally BioxCell^®^. Membrane permeability was shown to be better in samples, using Andromed^®^, followed by samples using Tris-EY and, finally, BioxCell^®^ samples. As for sperm viability analysis, a higher percentage of spermatozoa with an intact plasma membrane was observed when the Tris-EY extender was used, followed by Andromed^®^ samples and finally BioxCell^®^ samples. Lower motility percentages were reported by Torres Hualla [40] (12.04% for Tris-EY and 11.32% for the skimmed milk extender) compared to those obtained in the present study. Also, the sperm viability using Tris-EY was 18.96%, and using skimmed milk was 14.2%. Banda et al. [36] using different extenders such as Tris, Tea, and skimmed milk for alpaca sperm cryopreservation in samples collected by an artificial vagina, obtained motility percentages of 14.0, 8.6, and 17.0, respectively. In the same study, the results obtained for plasma membrane permeability (HOST) were 17.0, 19.1, and 17.9%, and viability was 32.6, 26.3, and 27.2%, respectively. These results were similar to those obtained in the present study, probably due to the similarity of the extender composition, such as Tris and Tes. Ordoñez et al. [41] carried out sperm collection using electroejaculation methods for alpaca sperm pellet cryopreservation using Andromed^®^- and Tris-obtaining motilities post-thawing of 0.00 and 11.08%, respectively. This was probably due to the type of sperm collection method (electroejaculation), which has the disadvantage of having different contaminants, such as urine debris, which can be harmful to sperm cells [39]. Overall, in the present study, a low population of physiologically functional spermatozoa was observed for BioxCell^®^ and an average for Andromed^®^ and Tris-EY extenders. This was maybe due to changes in sperm morphology and mitochondrial mass damage after the sperm freezing-thawing process. This may also be influenced by the sperm cryopreservation method since, in the present study, sperm cryopreservation was carried out using a curve of −20 °C/min and reaching a freezing temperature of −120 °C, which is apparently the appropriate methodology for camelids [20]. Some authors reported that sperm plasma membranes with a higher polyunsaturated fatty acids content are more sensitive to damage induced by lipid peroxidation [38]. Moreover, this kind of plasma membrane is more sensitive to damage by reactive oxygen species (ROS), which results in decreased sperm motility, probably due to the rapid loss of intracellular ATP, leading to axoneme damage and decreased sperm viability, among other consequences [42].

In the present study, the observed results indicate that there was similarity between the Tris-EY and Andromed^®^ extenders in most of the sperm quality parameters except for progressive motility during the equilibration phase at 5 °C, where similarities were observed among the three extenders for llama sperm collected from the vas deferens. Using electroejaculation methods, Choez et al. [43] evaluated the sperm motility and plasma membrane permeability (HOST) during the first hour of the cooling process and observed a motility of 50.93%, which produced similar results to the present study using the Tris-EY extender. Other motility parameters, such as total motility, were higher than those reported by other authors [44]. However, the results were similar when using the BioxCell^®^ extender [45]. On the other hand, progressive motility was lower in the present study and slightly higher when the Tris-EY extender was used, probably due to the fact that egg yolk improves progressive motility, as mentioned by Fumuso et al. [46]. Moreover, the results were slightly better than those obtained in the present study, for example, those reported for viability percentages using egg yolk-based extenders [31]. However, in the present study, in all extender groups, a decrease in viability of just 8 to 15% was observed compared to the collection timepoint, mainly due to the action of egg yolk (Tris-EY) and lecithins (Andromed^®^ and BioxCell^®^), since these components have the main function of maintaining membrane stability during the cooling process. In addition, these components have a high percentage of cholesterol that maintains the structure of the sperm plasma membrane, facilitating the adhesion of several molecules that protect the plasma membrane at low temperatures (cold shock), although in camelids, this phenomenon has not yet been well studied. It has been described that this process can alter the sperm membrane cholesterol/phospholipid ratio; however, the use of egg yolk-based extenders could be variable due to their composition of fatty acids, phospholipids, cholesterol and lipoproteins [11,15,17,47,48]. The slight loss of motility and other parameters indicated that spermatozoa obtained from the vas deferens were resistant to cold shock when in contact with extenders containing Tris-EY as well as those that did not contain egg yolks such as Andromed^®^ and BioxCell^®^. Furthermore, it has been reported that the process of a spontaneous or induced acrosome reaction occurs after the equilibrium phase in camelids [35,38,49]. Other recent studies in llamas have shown that the cooling process affects the sperm organelles, resulting in acrosome damage, mitochondrial loss, and the disorganization of axoneme and periaxoneme structures, increasing the level of reactive oxygen species (ROS) [45]. Finally, less binding at the oviduct level has been observed using refrigerated and frozen-thawed spermatozoa compared to fresh spermatozoa [31].

Regarding the objective motility parameters, most of the evaluated traits coincided with those determined by the subjective motility assessment (e.g., total motility), with higher values in samples using Andromed^®^, followed by Tris-EY and finally BioxCell^®^ before and after cryopreservation process. The kinetic parameters related to different types of velocities were variable and corresponded to the motility obtained before sperm pre-freezing and post-thawing. On the other hand, parameters such as WOB, which is related to the ratio of the average trajectory velocity over curvilinear velocity expressed as a percentage, and LIN, which shows the ratio between the straight trajectory and the average sperm trajectory, were similar among extender samples. Similarly, the same was observed for STR, which was quite high in all samples analyzed regardless of the extender used. In summary, all the kinetic variables related to indices were similar, while those with values directly related to the type of movement had differences among different extender samples used in sperm obtained from the vas deferens in llamas. VCL, VSL, and VAP parameters were shown to be higher when using Andromed^®^ compared to the Tris-EY extender, especially in thawed spermatozoa, which had higher velocity results (Andromed^®^) probably due to the soy lecithin content as opposed to egg yolk containing the Tris extender. This difference could be related to the higher proportion of low-density lipoproteins (LDLs) that provide greater sperm plasma membrane stability [17]. However, in egg yolk-containing extenders, such as Triladyl, interesting results have been obtained in other mammalian species with regard to STR, LIN, ALH, and BCF parameters, which were characterized by an efficient flagellar structure, ATP production, and consequently, the higher frequency of the sperm tail beating movement (BCF) [50].

The health concerns related to the use of animal proteins in sperm extenders have led to the development of alternative extenders for in vitro and in vivo use. The sperm samples diluted in the plant-based (soy lecithin-based) extender Andromed^®^ resulted in better sperm quality and in vitro fertility compared to Tris-EY and BioXcell. Overall, all sperm quality parameters improved using the Andromed^®^ extender, including objective and subjective motility, which is one of the most important parameters associated with semen fertilizing capacity. In other words, Andromed^®^ was more effective at preserving sperm head and flagellar structures, stimulating ATP production, and improving fertility capacity. Regarding the results obtained in the present study using cryopreserved spermatozoa and the in vitro fertilization rates using oocytes obtained from the slaughterhouse, the Andromed^®^-derived results were similar to those obtained using epididymal cryopreserved seminal samples from alpacas [51]. Therefore, plant-based extenders are as effective at protecting spermatozoa during the cryopreservation process as egg yolk extenders. Another alpaca study showed that the in vitro fertilization rates were similar to those obtained in the present study, with the difference that non-cryopreserved spermatozoa were used [52]. However, there were studies in alpacas where higher fertilization rates have been observed compared to those reported in the present study [23]. The main findings of the present study in relation to fertility were that (1) vas deferens-derived sperm diluted in Tris-EY and Andromed^®^ resulted in similar fertilization rates in both fresh and cryopreserved samples; (2) vas deferens-derived sperm diluted in BioXcell resulted in reduced sperm quality and in vitro fertilization rates compared to Tris-EY- and Andromed^®^-diluted samples in both fresh and cryopreserved samples; (3) the temperature fluctuation during the cryopreservation process was detrimental for sperm quality and in vitro fertilization rates in all samples irrespective of the extender used, with BioXcell^®^-derived samples being the most affected. In South American camelids, in vitro fertilization studies are very scarce, and the results are still quite low compared to other species, such as bovines, where much higher fertilization rates were reported [25,26,53]. However, the classical sperm quality parameters studied, such as total motility, progressive motility, oscillatory motility, plasma membrane integrity, and permeability, among others, and other studies performed with computerized equipment (CASA) showed better IVF results with the use of the commercial extender Andromed^®^ and also the Tris-EY extender [25,26]. The reduction in fertility associated with BioXcell^®^ could indicate that this extender would be less tolerant to temperature fluctuations, and thus, it would not be a suitable substitute for an egg yolk extender such as Tris-EY. When the semen temperature fluctuates, it triggers several morphological membrane changes, compacting or relaxing the packing of the phospholipid bilayer and causing membrane destabilization, resulting in a deleterious effect on sperm function and fertility. Finally, it should be noted that due to the differences obtained in the in vitro fertilization results, more studies are needed in order to increase reproductive efficiency through the use of Assisted Reproductive Technologies (ARTs) in South American camelids in order to obtain in vitro-produced embryos to be transferred [25]. For example, sperm metabolic activity is inversely related to extended sperm survival and during the sperm cryopreservation process, there is an increase in oxidative stress due to the production of reactive oxygen species (ROS). The addition of components, such as citric acid and glycerol, plays a role in reducing the levels of peroxide generated in the storage media. Moreover, modifying the percentage of egg yolk or lecithin could also be effective in reducing sperm metabolic activity and the detrimental effects derived from ROS, which are linked to a cell-aging effect and lead to an apoptotic stage losing the fertility capacity.

## 5. Conclusions

The sperm quality and in vitro fertilization rates of fresh and cryopreserved spermatozoa collected from llama (*Lama glama*) vas deferens were influenced by the extender used during the cryopreservation process. In general, Andromed^®^ showed slightly better results post-thawing regarding acrosome membrane integrity, plasma membrane permeability, sperm kinetic parameters, and in vitro fertilization rates when using sperm samples obtained from llama (*Lama glama*) vas deferens. Although in vitro fertilization rates were similar to those obtained in other camelid species, further research is needed to understand the effects of different preservation extenders in order to improve the in vitro fertilization rates from sperm samples obtained from llama (*Lama glama*) vas deferens.

## Figures and Tables

**Table 1:** Sperm morphological defects (%) in fresh (F) and cryopreserved (C) sperm samples obtained from llama (*Lama glama*) vas deferens using different extenders.

Morphological Parameter	Cryopreservation Extender		
	Tris-EY	Andromed^®^	BioxCell^®^
Cytoplasmic droplet _F_ (%)	6.27 ± 2.69 ^aA^	10.58 ± 2.49 ^aA^	8.53 ± 1.91 ^aA^
Cytoplasmic droplet _C_ (%)	6.37 ± 3.49 ^aA^	15.58 ± 2.76 ^aA^	8.37 ± 3.91 ^bA^
Macrocephalia _F_ (%)	0.21 ± 0.06 ^abA^	0.08 ± 0.02 ^aA^	0.41 ± 0.20 ^bA^
Macrocephalia _C_ (%)	0.31 ± 0.16 ^aA^	0.18 ± 0.04 ^bB^	0.51 ± 0.19 ^aA^
Fusiform head _F_ (%)	0.10 ± 0.01 ^aA^	0.09 ± 0.02 ^aA^	0.11 ± 0.03 ^aA^
Fusiform head _C_ (%)	7.46 ± 1.45 ^aB^	6.69 ± 1.59 ^bB^	7.71 ± 2.09 ^aB^
Microcephalia _F_ (%)	0.41 ± 0.33 ^aA^	0.39 ± 0.19 ^aA^	0.41 ± 0.33 ^aA^
Microcephalia _C_ (%)	2.41 ± 0.23 ^aB^	2.39 ± 0.34 ^aB^	2.17 ± 0.54 ^aB^
Detached head _F_ (%)	1.41 ± 0.52 ^aA^	0.37 ± 0.14 ^bA^	0.76 ± 0.25 ^abA^
Detached head _C_ (%)	11.12 ± 2.52 ^aB^	9.37 ± 1.42 ^aB^	12.56 ± 2.51 ^aB^
Midpiece defects _F_ (%)	30.21 ± 6.01 ^aA^	27.84 ± 5.52 ^aA^	28.97 ± 6.32 ^aA^
Midpiece defects _C_ (%)	29.10 ± 5.81 ^aA^	28.98 ± 6.77 ^aA^	30.55 ± 7.64 ^aA^
Bent/Coiled tail _F_ (%)	12.74 ± 1.99 ^aA^	11.37 ± 1.71 ^aA^	12.72 ± 1.58 ^aA^
Bent/Coiled tail _C_ (%)	16.32 ± 2.03 ^aA^	14.25 ± 2.93 ^aA^	17.01 ± 2.71 ^aB^
Double-tailed _F_ (%)	1.36 ± 0.29 ^aA^	0.62 ± 0.12 ^bA^	0.51 ± 0.21 ^bA^
Double-tailed _C_ (%)	1.86 ± 0.25 ^aA^	1.26 ± 0.21 ^aB^	1.51 ± 0.61 ^aB^
Detached tail _F_ (%)	5.43 ± 1.93 ^aA^	2.35 ± 0.37 ^bA^	3.05 ± 0.56 ^abA^
Detached tail _C_ (%)	9.09 ± 2.34 ^aB^	7.87 ± 1.73 ^bB^	9.95 ± 2.98 ^aB^
Total defects _F_ (%)	59.14 ± 2.00 ^aA^	53.69 ± 3.40 ^aA^	55.47 ± 3.30 ^aA^
Total defects _C_ (%)	84.00 ± 3.08 ^aB^	86.50 ± 3.00 ^aB^	90.34 ± 3.22 ^aB^

Different superscript letters (^a,b^) in a row represent significant differences among cryopreservation extenders (*p* < 0.05). Different superscript letters (^A,B^) in a column represent significant differences before (F) and after the cryopreservation (C) process within the same morphological parameter (*p* < 0.05). F: fresh; C: cryopreserved.

**Table 2:** Acrosome membrane integrity (%) in fresh and cryopreserved sperm samples obtained from llama (*Lama glama*) vas deferens using different extenders.

Parameter	Cryopreservation Extender		
	Tris-EY	Andromed^®^	BioxCell^®^
Acrosome membrane integrity (AI) at 37 °C (%)	70.58 ± 1.02 ^aA^	64.88 ± 0.48 ^bA^	59.20 ± 0.49 ^cA^
Acrosome membrane integrity (AI) at 5 °C (%)	60.83 ± 0.61 ^aB^	55.35 ± 0.71 ^bB^	53.64 ± 0.42 ^bB^
Acrosome membrane integrity (AI) post-thawing (%)	23.63 ± 0.58 ^aC^	31.52 ± 0.31 ^bC^	9.97 ± 0.31 ^cC^

Different superscript letters (^a–c^) in a row represent significant differences among cryopreservation extenders (*p* < 0.05). Different superscript letters (^A–C^) in a column represent significant differences among cryopreservation steps within the same extender (*p* < 0.05).

**Table 3:** Plasma membrane integrity (viability; %) and plasma membrane permeability (HOST) using different extenders during the cryopreservation process in sperm samples obtained from llama (*Lama glama*) vas deferens.

Parameter	Cryopreservation Extender		
	Tris-EY	Andromed^®^	BioxCell^®^
Plasma membrane integrity (Viability) at 37 °C (%)	48.91 ± 5.55 ^aA^	65.78 ± 3.38 ^bA^	55.89 ± 1.27 ^abA^
Plasma membrane integrity (Viability) at 5 °C (%)	45.23 ± 3.77 ^aAB^	61.57 ± 2.37 ^bA^	50.64 ± 0.86 ^abA^
Plasma membrane integrity (Viability) post-thawing (%)	38.57 ± 1.52 ^aB^	29.05 ± 0.48 ^bB^	10.98 ± 0.32 ^cB^
Hypo-Osmotic Swelling test (HOST) at 37 °C (%)	69.79 ± 2.83 ^aA^	62.68 ± 1.19 ^bA^	57.01 ± 2.27 ^bA^
Hypo-Osmotic Swelling test (HOST) at 5 °C (%)	43.88 ± 6.54 ^aB^	56.63 ± 1.89 ^aB^	46.19 ± 1.25 ^aB^
Hypo-Osmotic Swelling test (HOST) post-thawing (%)	24.78 ± 4.11 ^aC^	30.73 ± 2.35 ^bC^	20.07 ± 4.65 ^cC^

Different superscript letters (^a–c^) in a row represent significant differences among cryopreservation extenders (*p* < 0.05). Different superscript letters (^A–C^) in a column represent significant differences among cryopreservation steps within the same extender (*p* < 0.05).

**Table 4:** Sperm motility parameters using different extenders during the cryopreservation process in sperm samples obtained from llama (*Lama glama*) vas deferens.

Motility Parameter	Cryopreservation Extender		
	Tris-EY	Andromed^®^	BioxCell^®^
Total Motility at 37 °C (%)	61.77 ± 3.00 ^aA^	65.56 ± 2.11 ^aA^	38.33 ± 0.63 ^bA^
Total Motility at 5 °C (%)	54.71 ± 3.61 ^aA^	55.90 ± 0.85 ^aB^	33.21 ± 0.88 ^bB^
Total Motility Post-Thawing (%)	26.54 ± 1.52 ^aB^	31.37 ± 0.57 ^bC^	9.17 ± 0.51 ^cC^
Progressive Motility at 37 °C (%)	29.00 ± 3.23 ^aA^	22.44 ± 1.67 ^aA^	18.59 ± 1.38 ^aA^
Progressive Motility at 5 °C (%)	23.12 ± 4.48 ^aA^	15.81 ± 0.82 ^aB^	15.01 ± 0.66 ^aA^
Progressive Motility Post-Thawing (%)	11.10 ± 1.23 ^aB^	7.24 ± 0.57 ^bC^	2.05 ± 0.09 ^cB^
Oscillatory Motility at 37 °C (%)	34.96 ± 1.17 ^aA^	37.20 ± 2.16 ^aA^	17.06 ± 1.00 ^bA^
Oscillatory Motility at 5 °C (%)	27.65 ± 2.57 ^aB^	32.52 ± 0.85 ^aB^	16.30 ± 0.99 ^bA^
Oscillatory Motility Post-Thawing (%)	14.01 ± 0.89 ^aB^	23.01 ± 0.80 ^bB^	6.24 ± 0.34 ^cB^
Circular Motility at 37 °C (%)	7.82 ± 1.30 ^aA^	5.96 ± 0.69 ^aA^	2.69 ± 0.12 ^aA^
Circular Motility at 5 °C (%)	6.26 ± 2.21 ^aA^	7.58 ± 1.23 ^aA^	1.89 ± 0.16 ^bB^
Circular Motility Post-Thawing (%)	1.43 ± 0.62 ^aB^	1.12 ± 0.20 ^aB^	1.19 ± 0.05 ^aC^

Different superscript letters (^a–c^) in a row represent significant differences among cryopreservation extenders. Different superscript letters (^A–C^) in a column represent significant differences among cryopreservation steps within the same extender (*p* < 0.05).

**Table 5:** Sperm kinetic parameters using different extenders before (F) and after cryopreservation (C) in sperm samples obtained from llama (*Lama glama*) vas deferens.

Kinetic Parameter	Cryopreservation Extender		
	Tris-EY	Andromed^®^	Bioxcell^®^
VCL _F_ (µm/s)	36.16 ± 1.41 ^aA^	34.76 ± 1.37 ^aA^	33.06 ± 2.67 ^aA^
VCL _C_ (µm/s)	14.17 ± 1.04 ^aB^	25.33 ± 1.94 ^bB^	15.33 ± 1.56 ^aB^
VSL _F_ (µm/s)	20.50 ± 0.87 ^aA^	20.63 ± 1.58 ^aA^	10.91 ± 1.94 ^bA^
VSL _C_ (µm/s)	7.57 ± 0.67 ^aB^	12.90 ± 1.08 ^bB^	9.03 ± 0.90 ^aA^
VAP _F_ (µm/s)	24.71 ± 1.46 ^aA^	24.52 ± 1.71 ^aA^	16.64 ± 1.82 ^bA^
VAP _C_ (µm/s)	10.00 ± 0.86 ^aB^	17.16 ± 1.38 ^bB^	10.50 ± 1.09 ^aB^
DCL _F_ (µm)	9.73 ± 0.64 ^aA^	8.55 ± 1.23 ^aA^	14.14 ± 1.78 ^bA^
DCL _C_ (µm)	4.73 ± 0.34 ^aB^	8.40 ± 0.88 ^bA^	4.67 ± 0.51 ^aB^
DSL _F_ (µm)	2.82 ± 2.14 ^aA^	2.56 ± 1.31 ^aA^	4.04 ± 0.52 ^aA^
DSL _C_ (µm)	1.55 ± 0.14 ^aA^	2.33 ± 0.16 ^bA^	1.60 ± 0.19 ^aB^
DAP _F_ (µm)	4.67 ± 0.81 ^aA^	4.24 ± 0.66 ^aA^	6.76 ± 0.86 ^bA^
DAP _C_ (µm)	2.77 ± 0.21 ^aB^	4.45 ± 0.46 ^bA^	2.30 ± 0.28 ^aB^
ALH _F_ (µm)	0.36 ± 0.07 ^aA^	0.33 ± 0.08 ^aA^	0.49 ± 0.09 ^aA^
ALH _C_ (µm)	0.22 ± 0.02 ^aB^	0.32 ± 0.04 ^aA^	0.18 ± 0.03 ^bB^
BCF _F_ (Hz)	2.78 ± 0.81 ^aA^	2.42 ± 0.24 ^aA^	4.91 ± 0.27 ^bA^
BCF _C_ (Hz)	0.82 ± 0.11 ^aB^	2.02 ± 0.24 ^bB^	0.57 ± 0.13 ^aB^
HAC _F_ (rad)	0.09 ± 0.00 ^aA^	0.08 ± 0.00 ^aA^	0.10 ± 0.04 ^aA^
HAC _C_ (rad)	0.04 ± 0.00 ^aB^	0.05 ± 0.00 ^aB^	0.03 ± 0.00 ^bB^
WOB _F_ (VAP/VCL)	0.68 ± 0.01 ^aA^	0.71 ± 0.07 ^aA^	0.50 ± 0.09 ^aA^
WOB _C_ (VAP/VCL)	0.70 ± 0.01 ^aA^	0.68 ± 0.02 ^aA^	0.68 ± 0.02 ^aB^
LIN _F_ (VSL/VCL)	0.57 ± 0.02 ^aA^	0.59 ± 0.04 ^aA^	0.33 ± 0.03 ^aA^
LIN _C_ (VSL/VCL)	0.50 ± 0.02 ^aB^	0.51 ± 0.04 ^aA^	0.60 ± 0.02 ^bB^
STR _F_ (VCL/VAP)	0.83 ± 0.02 ^aA^	0.84 ± 0.09 ^aA^	0.66 ± 0.00 ^bA^
STR _C_ (VCL/VAP)	0.77 ± 0.02 ^aB^	0.75 ± 0.03 ^aA^	0.86 ± 0.00 ^bB^

Different superscript letters (^a,b^) in a row represent significant differences among cryopreservation extenders (*p* < 0.05). Different superscript letters (^A,B^) in a column within the same parameter represent significant differences within the same extender (*p* < 0.05). Curved line velocity (VCL, μm s^−1^), straight-line velocity (VSL, μm s^−1^), average pathway velocity (VAP, μm s^−1^), curved line distance (DCL, μm), straight line distance (DSL, μm), average path distance (DAP, μm), amplitude lateral head displacement (ALH, μm), beat cross frequency (BCF, Hz), head activity (HAC, rad), wobble (WOB, ratio), linearity (LIN, VSL/VCL ratio), and straightness (STR, VSL/VAP ratio). F: fresh; C: cryopreserved.

**Table 6:** In vitro fertilization rates (%) using different extenders from fresh and cryopreserved sperm samples obtained from llama (*Lama glama*) vas deferens.

Type of Sperm	Sperm Extender		
	Tris-EY Fertilized Oocytes/ Total Oocytes Submitted to IVF (%)	Andromed^®^ Fertilized Oocytes/ Total Oocytes Submitted to IVF (%)	BioxCell^®^ Fertilized Oocytes/ Total Oocytes Submitted to IVF (%)
Fresh	47/149 (31.54) ^aA^	51/149 (34.23) ^aA^	36/150 (24.00) ^bA^
Frozen-thawed	40/180 (22.22) ^aB^	44/179 (24.58) ^aB^	30/180 (16.66) ^bB^

Different superscript letters (a,b) in a row represent significant differences among sperm extenders within the same type of sperm (*p* < 0.05). Different superscript letters (A,B) in a column within the same extender represent significant differences between fresh and cryopreserved sperm (*p* < 0.05). Total No. oocytes (fresh): 448; Total No. oocytes (frozen-thawed): 539.

## Data Availability

All data generated or analyzed during this study are included in this article.
